# Calderón’s Method with a Spatial Prior for 2-D EIT Imaging of Ventilation and Perfusion

**DOI:** 10.3390/s21165635

**Published:** 2021-08-21

**Authors:** Kwancheol Shin, Jennifer L. Mueller

**Affiliations:** 1 Department of Mathematics, Chungbuk National University, Cheongju 28644, Korea; 2 Department of Mathematics and School of Biomedical Engineering, Colorado State University, Fort Collins, CO 80523, USA; mueller@math.colostate.edu

**Keywords:** electrical impedance tomography, inverse problems, Calderón’s method, pulmonary imaging

## Abstract

Bedside imaging of ventilation and perfusion is a leading application of 2-D medical electrical impedance tomography (EIT), in which dynamic cross-sectional images of the torso are created by numerically solving the inverse problem of computing the conductivity from voltage measurements arising on electrodes due to currents applied on electrodes on the surface. Methods of reconstruction may be direct or iterative. Calderón’s method is a direct reconstruction method based on complex geometrical optics solutions to Laplace’s equation capable of providing real-time reconstructions in a region of interest. In this paper, the importance of accurate modeling of the electrode location on the body is demonstrated on simulated and experimental data, and a method of including a priori spatial information in dynamic human subject data is presented. The results of accurate electrode modeling and a spatial prior are shown to improve detection of inhomogeneities not included in the prior and to improve the resolution of ventilation and perfusion images in a human subject.

## 1. Introduction

Electrical impedance tomography has been demonstrated to be a promising technique for pulmonary imaging at the bedsides of critically ill patients and for those with chronic lung disease. Recent survey articles on clinical applications of EIT include [1,2] and the reader is also referred to the articles [3,4,5,6,7,8,9] for an introduction.

Calderón’s method is a direct reconstruction method based on complex geometrical optics (CGO) solutions to Laplace’s equation. The method was outlined by Calderón in his 1980 paper *On an inverse boundary value problem* [10], in which he proved that the linearized problem has a unique solution and proposed a direct method of reconstruction. The method can be viewed as a linearization of the D-bar method [11,12] based on the 1996 global uniqueness proof by Nachman [13]. While the D-bar method makes use of a certain type of nonlinear Fourier transform known as the *scattering transform* as an intermediate function in the solution of a $\bar{\partial }$ (D-bar) equation to directly compute the conductivity, Calderón’s method relies on computing the inverse Fourier transform of a bilinear form involving the data and CGO solutions to obtain the conductivity. Both methods require knowledge of the Dirichlet-to-Neumann or voltage-to-current density map. Calderón’s method was first implemented numerically in [14] for experimental EIT data collected on a saline-filled tank and on a healthy human subject reconstructed on a circular domain. In [15], this was extended to noncircular but symmetric domains. A real-time implementation in 2-D on subject-specific domains was presented in [16,17]. A “higher-order” Calderón’s method and a method of including a spatial prior was presented in [12] with demonstrated results from data collected on a circular saline-filled tank with agar and vegetable targets. A 3-D Calderón-based method was provided in [18] in the planar geometry and [19] for a cylinder.

In this paper, an implementation in which the exact locations of the electrodes are known is provided. Previous work on a subject-specific domain [16,17] assumed that the electrodes were equally spaced in their angular positions around the boundary, leading to some simplification in the computational formulas. A further novel contribution of this paper is a method of including a static prior derived from CT scans and a preliminary reconstruction to compute dynamic images of ventilation and perfusion. The relevant medical application for this scenario would be a case when a CT scan and EIT scan are performed upon hospitalization, and then the patient is monitored with EIT for pulmonary complications, such as a pneumothorax or pleural effusion, both of which are conditions that may develop, for example, in patients with acute respiratory distress syndrome (ARDS) receiving mechanical ventilation.

The use of a spatial prior to improve the resolution of reconstructed EIT images has proved to be effective in iterative methods [20,21,22,23,24,25,26,27,28,29] and in the D-bar method [30,31,32,33]. In the D-bar method, the scattering transform computed from the data is appended with a scattering transform computed from a conductivity prior. In [34], a statistical approach using the Schur complement was used to construct a prior for the D-bar method. In [12], the Fourier transform of the prior conductivity distribution is appended to the bilinear form before inverting. Dynamic images of the human chest prove more challenging in the construction of a prior conductivity distribution. In [35] a dynamic prior was constructed for the D-bar method applied to ventilation data from inspiratory and expiratory CT scans of the patient. In this work, a static prior is constructed by segmenting a CT scan of a patient with cystic fibrosis, and the conductivity values for the lung, heart, and background are assigned from a preliminary reconstruction with Calderón’s method.

Since Calderon’s method has attracted attention as a direct real-time reconstruction method, we aim to show its potential for practical implementation, which has both advantages and shortcomings. Other methods may result in higher resolution images, some with significant trade-offs, such as computation time. Gauss–Newton methods require a forward model for the iterative updates, which to date precludes real-time reconstruction. However, careful modeling also contributes to improvements in resolution. Including a prior further improves performance [20,21,22,23,24,25,26,28,29]. The D-bar method is a direct reconstruction method for which spatial priors have been included by appending the scattering transform computed from the measured data with the scattering transform of the prior. The D-bar method also can be implemented in real time, although Calderón’s method produces reconstructions even faster [16]. Shape reconstruction methods [36,37,38,39] have the advantage that they can incorporate geometry and prior information directly and preserve sharp edges, also reducing the computational burden of the full reconstruction problem, which also shows promise for lung imaging.

The paper is organized as follows. In Section 2, the mathematical model of EIT is provided. In Section 3, Calderón’s method and our numerical implementation with exact electrode modeling and the inclusion of a spatial prior are presented. The results of the method on experimental data collected on a chest-shaped tank, simulated data with several simulated pulmonary pathologies, and human data collected on a patient are provided in Section 4. The conclusion is presented in Section 5.

## 2. Background

### Modeling of EIT

Let $\gamma \left(x\right)\geq \gamma_{0}>0$ be the electrical conductivity with a positive constant lower bound $\gamma_{0}$, u(x) be the electrical potential and $\Omega \in R^{2}$ be a bounded domain. The governing equation of EIT is

(1) ∇·γ(x)∇u(x)=0,x∈Ω.

The applied current density *j* on the boundary corresponds to the Neumann boundary condition

(2) $$\gamma \left(x\right)\frac{\partial u}{\partial \nu }\left(x\right)=j\left(x\right),\;\;x\in \partial \Omega ,$$

where ν is the outward normal vector to ∂Ω. We denote the voltage distribution on the boundary by *f* so that

(3) u(x)=f(x),x∈∂Ω,

is the corresponding Dirichlet boundary condition for (1). If γ(x) and one of the boundary conditions (2) or (3) are given, the forward problem is to solve for u(x) in Ω. The inverse problem is to find the unknown γ(x) from knowledge of the Neumann-to-Dirichlet (ND) map,

$$R_{\gamma }:\gamma \left(x\right)\frac{\partial u}{\partial \nu }\left(x\right)⟶u\left(x\right),\;x\in \partial \Omega ,$$

which is also called the current density-to-voltage map. However, in most of the mathematical literature, the theory is developed using the Dirichlet-to-Neumann (DN) map

(4) $$\Lambda_{\gamma }:u\left(x\right)⟶\gamma \left(x\right)\frac{\partial u}{\partial \nu }\left(x\right),\;x\in \partial \Omega ,$$

or voltage-to-current density map.

## 3. Methods

### 3.1. Calderon’s Method

For completeness, we summarize Calderón’s method from [10]. Assume that γ(x) is a small perturbation $\delta \left(x\right)\in L^{\infty }\left(\Omega \right)$ from a background conductivity of 1 so that γ(x)=1+δ(x). Calderón’s method uses a special type of harmonic function known as *complex geometrical optics (CGO)* solutions defined by

$$f\left(x;k,a\right)=e^{\pi i(k\cdot x)+\pi (a\cdot x)},\;\;\;g\left(x;k,a\right)=e^{\pi i(k\cdot x)-\pi (a\cdot x)},$$

where $k,a\;\in R^{2}$ are nonphysical frequency variables with |k|=|a|,k·a=0. Next, let $\omega_{i}$ be defined on Ω as

$$\omega_{i}=u_{i}+v_{i}\;\mathrm{in}\;\;\Omega ,\;\;i=1,2,$$

where $\omega_{1}=f$ and $\omega_{2}=g$ on ∂Ω, $u_{i}\in H^{1}\left(\Omega \right)$, and $v_{i}\in H_{0}^{1}\left(\Omega \right)$ for i=1,2. Here, $H^{1}\left(\Omega \right)$ denotes the Sobolev space of functions with one weak derivative in $L^{2}\left(\Omega \right)$ and $H_{0}^{1}\left(\Omega \right)$ the Sobolev space $H^{1}\left(\Omega \right)$ with trace zero [40]. Then, by (1) and integration by parts,

(5) $$\int_{\partial \Omega }f\left(x,k\right)\Lambda_{\gamma }g\left(x,k\right)ds\left(x\right)=-2\pi^{2}{\left|k\right|}^{2}\int_{\Omega }\gamma \left(x\right)\exp\left[2\pi ix\cdot k\right]dx+\tilde{R}\left(k\right),$$

where $\tilde{R}\left(k\right)$ is considered to be a remainder term. Rearranging terms results in

$$\int_{\Omega }\gamma \left(x\right)\exp\left[2\pi ix\cdot k\right]dx=-\frac{1}{2\pi^{2}{\left|k\right|}^{2}}\int_{\partial \Omega }f\left(x,k\right)\Lambda_{\gamma }g\left(x,k\right)ds\left(x\right)+\hat{R}\left(k\right),$$

where $\hat{R}\left(k\right)=\frac{\tilde{R}\left(k\right)}{2\pi^{2}{\left|k\right|}^{2}}$. Assuming γ(x) is constant outside of Ω, the left-hand side is the Fourier transform of $\gamma \chi_{\Omega }$, which we denote by $\hat{\gamma }\left(k\right)$. We denote the first term on the right-hand side by

(6) $${\hat{F}}_{\gamma }^{\mathrm{abs}}\left(k\right)\equiv -\frac{1}{2\pi^{2}{\left|k\right|}^{2}}\int_{\partial \Omega }f\left(x,k\right)\Lambda_{\gamma }g\left(x,k\right)ds\left(x\right).$$

Calderón showed that when ${\left||\delta \left(x\right)|\right|}_{L^{\infty }\left(\Omega \right)}$ is small, $|\hat{R}\left(k\right)|$ is small for small values of |k|. Since $\gamma \left(x\right)\chi_{\Omega }$ is zero outside of Ω, $|\hat{\gamma }\left(k\right)|$ has to decrease to zero as |k| becomes larger. Therefore, by multiplying a mollifying function $\hat{\eta }\left(k\right)$ such that $\hat{\eta }\left(0\right)=1$, $\hat{\eta }\in C^{\infty }$ and that decreases fast to zero as |k| becomes larger, ${\hat{F}}_{\gamma }^{\mathrm{abs}}\hat{\eta }$ approximates $\hat{\gamma }$, and it is shown in [10] that the inverse Fourier transform of $\hat{R}\left(k\right)\hat{\eta }\left(k\right)$ is negligible when ${\left||\delta |\right|}_{L^{\infty }}$ is small. The use of the mollification is equivalent to applying a low-pass filter to ${\hat{F}}_{\gamma }^{\mathrm{abs}}$. This low-pass filtering can be carried out alternatively by truncating ${\hat{F}}_{\gamma }^{\mathrm{abs}}\left(k\right)$ into a disk of radius $R_{1}$. We call $R_{1}$ the truncation radius. Therefore, we obtain an approximation of γ(x) by

(7) $$\gamma^{\mathrm{abs}}\left(x\right)\equiv \int_{\left|k\right|\leq R_{1}}{\hat{F}}_{\gamma }^{\mathrm{abs}}\left(k\right)\hat{\eta }\left(k/t\right)e^{-2\pi ix\cdot k}dk,$$

where $\hat{\eta }\left(k/t\right)$ is a mollifying function with $\hat{\eta }\left(0\right)=1$ and *t* is the mollfication parameter. We call the reconstruction from (7) *the absolute image of γ*.

Let $\Lambda_{1}$ denote the Dirichlet-to-Neumann map corresponding to a constant conductivity distribution of γ=1. By subtracting ${\hat{F}}_{1}^{\mathrm{abs}}\left(k\right)$ from ${\hat{F}}_{\gamma }^{\mathrm{abs}}\left(k\right)$, defined in Equation (6), we define

(8) $${\hat{F}}_{\mathrm{hom}}^{\mathrm{diff}}\left(k\right)\equiv -\frac{1}{2\pi^{2}{\left|k\right|}^{2}}\int_{\partial \Omega }f\left(x,k\right)\left(\Lambda_{\gamma }-\Lambda_{1}\right)g\left(x,k\right)ds\left(x\right),$$

Inverting (8) on the truncated domain $\left|k\right|\leq R_{1}$ results in an approximation of the perturbation δ(x), which we denote by

(9) $$\delta_{\mathrm{hom}}^{\mathrm{diff}}\left(x\right)\equiv \int_{\left|k\right|\leq R_{1}}{\hat{F}}_{\mathrm{hom}}^{\mathrm{diff}}\left(k\right)\hat{\eta }\left(k/t\right)e^{-2\pi ix\cdot k}dk,$$

and call *the difference image of δ with respect the homogeneous data*.

Note, however, that the computation of the difference image requires knowledge of $\Lambda_{1}$, which in most practical applications such as in medical imaging, cannot be measured. On the other hand, the action of $\Lambda_{1}$ on g(x,k) can be computed analytically,

$$\int_{\Omega }f\left(x,k\right)\Lambda_{1}g\left(x,k\right)dx=-2\pi^{2}{\left|k\right|}^{2}\int_{\Omega }e^{2\pi ik\cdot x}dx,$$

and Equation (8) can be replaced by

(10) $${\hat{F}}_{\delta }^{\mathrm{abs}}\left(k\right)\equiv -\frac{1}{2\pi^{2}{\left|k\right|}^{2}}\int_{\partial \Omega }f\left(x,k\right)\Lambda_{\gamma }g\left(x,k\right)ds\left(x\right)-\int_{\Omega }e^{2\pi ik\cdot x}dx.$$

We define

(11) $$\delta^{\mathrm{abs}}\left(x\right)\equiv \int_{\left|k\right|\leq R_{1}}{\hat{F}}_{\delta }^{\mathrm{abs}}\left(k\right)\hat{\eta }\left(k/t\right)e^{-2\pi ix\cdot k}dk,$$

and call it the *the absolute image of δ*.

While one can compute γ(x) directly by using (7), the use of (9) or (11) avoids the Gibbs phenomena near ∂Ω, which is introduced by the sharp discontinuity in $\gamma \left(x\right)\chi_{\Omega }$ along ∂Ω. Under the assumption that δ(x) is zero and therefore flat near ∂Ω, there are no Gibbs phenomena in the reconstructed images. Gibbs phenomena in the absolute images of γ can be observed in [12,14].

Another possibility that is particularly relevant to medical imaging is to choose one frame in a dynamic sequence of images as a reference image. For ventilatory images, this is often the frame corresponding to maximal exhalation and for perfusion images, this could correspond to peak systole, peak diastole, or a time point in between. Denoting the Dirichlet-to-Neumann map for the reference frame by $\Lambda_{\gamma_{\mathrm{ref}}}$, subtracting ${\hat{F}}_{\gamma_{\mathrm{ref}}}^{\mathrm{abs}}$ for $\gamma_{\mathrm{ref}}$ from Equation (6) results in

(12) $${\hat{F}}_{\mathrm{ref}}^{\mathrm{diff}}\left(k\right)\equiv -\frac{1}{2\pi^{2}{\left|k\right|}^{2}}\int_{\partial \Omega }f\left(x,k\right)\left(\Lambda_{\gamma }-\Lambda_{\gamma_{\mathrm{ref}}}\right)g\left(x,k\right)ds\left(x\right),$$

Taking the Fourier transform to (12) in the region $\left|k\right|\leq R_{1}$, we obtain an approximation to $\gamma -\gamma_{\mathrm{ref}}$ which we will denote by $\delta_{\mathrm{ref}}^{\mathrm{diff}}\left(x\right)$ and call it *the difference image of δ with respect to the reference frame*. We will make clear whether we are using a homogeneous data as in (8) and (9) or a specific frame in a dynamic sequence of data as in (12) when we refer to *the difference image*.

### 3.2. Numerical Implementation

Let $\left\{T^{i}\right\}$ be a set of linearly independent current patterns, i=1,⋯,L−1, applied to *L* electrodes. For i=1,⋯,L−1,l=1,⋯,L, let $T_{l}^{i}$ denote the *i*th current pattern on the *l*th electrode and $V_{l}^{i}$ the measured voltage. By Kirchhoff’s law, the elements of $T^{i}$ must satisfy $\sum_{l=1}^{L}T_{l}^{i}=0$, and for a unique choice of ground we require $\sum_{l=1}^{L}V_{l}^{i}=0$. Let $t^{i}$ denote the normalized current $t^{i}=\left(T^{i}\right)/\left(|\right|T^{i}{\left|\right|}_{2})$ and $v^{i}$ the normalized voltage $v^{i}=\left(V^{i}\right)/\left(|\right|T^{i}{\left|\right|}_{2})$, where $\left|\right|T^{i}{\left|\right|}_{2}=\sqrt{\sum_{l=1}^{L}{\left(T_{l}^{i}\right)}^{2}}$. In this work, the adjacent current patterns were applied on the electrodes and are given by

$$\begin{array}{c} T_{l}^{i}=\left\{\begin{array}{cc} M, & l=i,\\ -M, & l=i+1\;\mathrm{mod}\;L,\\ 0, & \mathrm{otherwise}. \end{array}\right. \end{array}$$

We model the current density j(x) on the boundary by the gap model,

$$\begin{array}{c} j\left(x\right)=\left\{\begin{array}{cc} \frac{I_{l}}{A}, & x\in e_{l}\\ 0, & \mathrm{otherwise}, \end{array}\right. \end{array}$$

where $e_{l}$ denotes the *l*th electrode and *A* is the area of an electrode, which is assumed to be the same for all electrodes.

Assume the locations of the centers of each of the electrodes is known, and let ${x|}_{\partial \Omega }=\left(x_{1}\left(\theta \right),x_{2}\left(\theta \right)\right)=r\left(\theta \right)\left(\cos\theta ,\sin\theta \right)$ be a parameterization of the boundary of Ω by θ, and the line element $ds=\sqrt{{\left(x_{1}^{\prime }\right)}^{2}+{\left(x_{2}^{\prime }\right)}^{2}}d\theta =\sqrt{r^{2}+r^{\prime 2}}d\theta .$ Denote the angle that the center of the *l*th electrode makes with a reference point chosen to be 0 degrees by ${\left(\theta_{l}\right)}_{l=1}^{l=L}$, and the angle between the center of the *l*th and l+1st electrode by $\Delta \theta_{l}=\theta_{l+1}-\theta_{l}$. For functions r,s of θ such that $r,s:R^{L}\to R$, let ${\left(r\left(\cdot \right),s\left(\cdot \right)\right)}_{L}$ denote the discrete inner product defined by ${\left(r\left(\cdot \right),s\left(\cdot \right)\right)}_{L}=\sum_{l=1}^{L}r\left(\theta_{l}\right)s\left(\theta_{l}\right).$

Expand $f(x_{l},k)$ and $g(x_{l},k)$ with respect to the normalized current patterns and the measured voltages as follows:

(13) $$\begin{array}{ccc} f(x_{l},k) & = & \sum_{i=1}^{L-1}f_{k}^{i}t_{l}^{i}, \end{array}$$

(14) $$\begin{array}{ccc} g(x_{l},k) & = & \sum_{j=1}^{L-1}g_{k,\gamma }^{j}v_{l}^{j,\gamma }, \end{array}$$

and let $f_{k}={\left\{f_{k}^{i}\right\}}_{i=1}^{L-1}$ and $g_{k,\gamma }={\left\{g_{k,\gamma }^{j}\right\}}_{j=1}^{L-1}$ be the coefficient vectors, where the subscripts *k* and γ indicate the dependence of the coefficients on the variable *k* and the conductivity γ. Since the applied normalized current patterns and the measured voltages are not orthogonal in general, in order to compute $f_{k}$ and $g_{k,\gamma }$, we need to solve systems of linear equations as follow. Taking the inner product with $v^{i,\gamma }\;\left(i=1,2,...,L-1\right)$ on both sides of (14), we obtain $V_{\gamma }g_{k,\gamma }=c_{k,\gamma },$ where $V_{\gamma }\left(i,j\right)={\left(v^{i,\gamma },v^{j,\gamma }\right)}_{L}$ and $c_{k,\gamma }\left(i\right)={\left(g\left(x_{l},k\right),v_{l}^{i,\gamma }\right)}_{L}$. Therefore, $g_{k,\gamma }=V_{\gamma }^{-1}c_{k,\gamma }$. Similarly, $f_{k}=T^{-1}d_{k}$, where $T\left(i,j\right)={\left(t^{i},t^{j}\right)}_{L}$ and $d_{k}\left(i\right)={\left(f\left(x_{l},k\right),t_{l}^{i}\right)}_{L}$. Now, with (13) and (14),

$$\begin{array}{cc} \int_{\partial \Omega }f\left(x,k\right)\Lambda_{\gamma }g\left(x,k\right)ds\left(x\right)\; & =\int_{0}^{2\pi }\sum_{i=1}^{L-1}f_{k}^{i}t^{i}\left(\theta \right)\left[\Lambda_{\gamma }\sum_{j=1}^{L-1}g_{k,\gamma }^{j}v^{j,\gamma }\left(\cdot \right)\right]ds\left(\theta \right)\\ \; & =\sum_{i=1}^{L-1}\sum_{j=1}^{L-1}f_{k}^{i}g_{k,\gamma }^{j}\int_{0}^{2\pi }t^{i}\left(\theta \right)\left[\Lambda_{\gamma }v^{j,\gamma }\left(\cdot \right)\right]ds\left(\theta \right)\\ \; & =\frac{1}{A}\sum_{i=1}^{L-1}\sum_{j=1}^{L-1}f_{k}^{i}g_{k,\gamma }^{j}\sum_{l=1}^{L}t_{l}^{i}t_{l}^{j}e_{w},\\ \; & =\frac{e_{w}}{A}f_{k}^{T}Tg_{k,\gamma }, \end{array}$$

where $e_{w}$ is the width of one electrode, where all are assumed to be equal, and $f_{k}^{T}$ is the transpose of $f_{k}$. Notice that the coefficients $f_{k}$ and $g_{k,\gamma }$ encode the information about the location of electrodes and therefore the boundary shape provided that we use the exact values of $f(x_{l},k)$ and $g(x_{l},k)$ in (13) and (14). From (8), (10), and (12), and by the similar calculation,

(15) $${\hat{F}}_{\mathrm{homo}}^{\mathrm{diff}}\left(k\right)=-\frac{e_{w}}{2\pi^{2}A{\left|k\right|}^{2}}f_{k}^{T}T\left(g_{k,\gamma }-g_{k,1}\right),$$

(16) $${\hat{F}}_{\delta }^{\mathrm{abs}}\left(k\right)=-\frac{e_{w}}{2\pi^{2}A{\left|k\right|}^{2}}f_{k}^{T}Tg_{k,\gamma }-\int_{\Omega }e^{2\pi ik\cdot x}dx,$$

and

(17) $${\hat{F}}_{\mathrm{ref}}^{\mathrm{diff}}\left(k\right)=-\frac{e_{w}}{2\pi^{2}A{\left|k\right|}^{2}}f_{k}^{T}T\left(g_{k,\gamma }-g_{k,\mathrm{ref}}\right).$$

The approximations $\delta_{\mathrm{homo}}^{\mathrm{diff}}$, $\delta^{\mathrm{abs}}$ and $\delta_{\mathrm{ref}}^{\mathrm{diff}}$ are computed by taking the inverse Fourier transform of (15)–(17), respectively. The inverse Fourier transform is computed for $\left|k\right|\leq R_{1}$, where $R_{1}$ is the truncation radius. This radius is unitless, and it depends on the scaling of the domain. If, for example, the maximum value of |x| is 180 mm, and we scale the domain to have a maximum of |x|=1; then, we rescale the truncation radius to $\frac{R_{1}}{180}$ Simpson’s quadrature rule is used to compute the integral of the inverse Fourier transform. Once we compute $\delta_{\mathrm{homo}}^{\mathrm{diff}}$, $\delta^{\mathrm{abs}}$ and $\delta_{\mathrm{ref}}^{\mathrm{diff}}$, we add the background conductivity to those to get the conductivity distributions $\gamma_{\mathrm{homo}}^{\mathrm{diff}}$, $\gamma^{\mathrm{abs}}$ and $\gamma_{\mathrm{ref}}^{\mathrm{diff}}$, respectively.

### 3.3. Calderón’s Method with a Spatial Prior

In [12], a method of including a spatial prior to Calderón’s method is introduced. We implement the method in this paper. We assume that we have a priori knowledge for the boundary of organs and the approximate regional conductivity for each organ. We denote this a priori conductivity distribution by $\delta_{\mathrm{pr}}\left(x\right)$. By adding a high-pass filtered prior $\int_{R_{1}\leq \left|k\right|\leq R_{2}}{\hat{\delta }}_{\mathrm{pr}}\left(k\right)\hat{\eta }\left(k/t\right)e^{-2\pi ik\cdot x}dk$ to the reconstructions obtained from (11) and (12), we define

(18) $$\delta_{\mathrm{pr}}^{\mathrm{abs}}\left(x\right)=\delta^{\mathrm{abs}}\left(x\right)+\int_{\mathrm{pri}}{\hat{\delta }}_{\mathrm{pr}}\left(k\right)\hat{\eta }\left(k/t\right)e^{-2\pi ik\cdot x}dk,$$

and

(19) $$\delta_{\mathrm{pr}}^{\mathrm{diff}}\left(x\right)=\delta^{\mathrm{diff}}\left(x\right)+\int_{R_{1}\leq \left|k\right|\leq R_{2}}{\hat{\delta }}_{\mathrm{pr}}\left(k\right)\hat{\eta }\left(k/t\right)e^{-2\pi ik\cdot x}dk,$$

for some constant $R_{2}$, where $\delta^{\mathrm{diff}}$ is either $\delta_{\mathrm{homo}}^{\mathrm{diff}}$ or $\delta_{\mathrm{ref}}^{\mathrm{diff}}$ depending on the applications.We define $\gamma_{\mathrm{pr}}^{\mathrm{abs}}$ and $\gamma_{\mathrm{pr}}^{\mathrm{diff}}$ by adding the background conductivity to (18) and (19), respectively.

## 4. Experimental Results

### 4.1. Chest Shaped Tank Data

In this section, the improvement in resolution when correct electrode positions are included in the algorithm is demonstrated. In this section, no spatial prior is used in the reconstruction algorithm. Data was collected on a chest-shaped tank of with circumference 1.016 m, simulating the shape of a human subject. The tank was filled with saline of conductivity of 0.2 S/m to a height of 0.0204 m, and three inclusions featuring two lungs of conductivity 0.09 S/m and the heart of conductivity 0.45 S/m. The width of the electrodes was 0.254 m. From the center of the tank, the electrodes are spaced nonuniformly in angle. For the homogeneous data for the difference images of δ, a data set was collected with only saline in the tank. The data was taken with the Active Complex Electrode (ACE1) system (see, [41,42]) in the EIT lab at Colorado State University. The frequency of the system was 125 kHz and the current amplitude was 3.3 mA.

**Figure 1:**
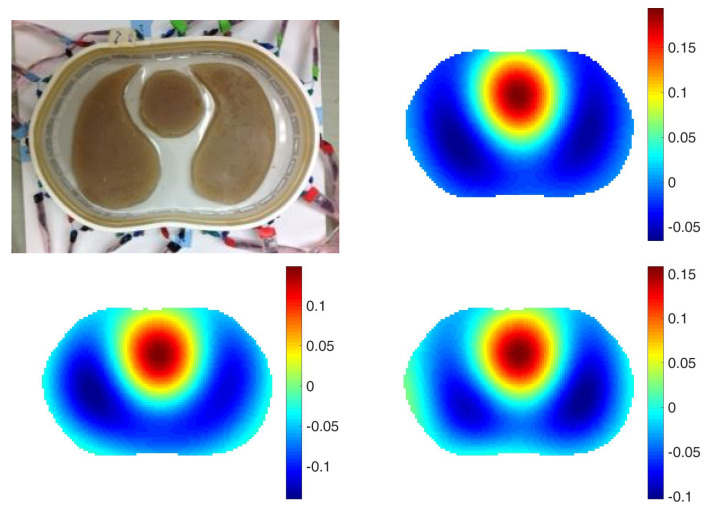
Top left: The chest shaped tank filled with saline bath and inclusions simulating two low conductive lungs and the high conductive heart. Top right: A difference image without modeling the location of electrodes. Bottom left: A difference image with modeling the location of electrodes. Bottom right: An absolute image with modeling the location of electrodes. The L2 norms of the differences between those reconstructions and the ground truth are 0.1128, 0.0973 and 0.0981 in order.

See Figure 1 for a photo of the experimental configuration and reconstructions of a difference image of $\delta_{\mathrm{homo}}^{\mathrm{diff}}$ with the algorithm suggested in [16] in which the location of electrodes is assumed to be spaced uniformly in angle (upper right). Without correct electrode modeling, absolute images were unattainable. The truncation radius is 1.2 for all image. The lower left image in Figure 1 is a reconstruction of $\delta_{\mathrm{homo}}^{\mathrm{diff}}$ with correct electrode modeling suggested in this paper. The lower right image is an absolute image, $\delta^{\mathrm{abs}}$ with the same truncation radius. With correct electrodes modeling, the absolute image of δ is comparable to the difference image The L2 norms of the differences between those reconstructions and the ground truth are 0.1128, 0.0973 and 0.0981 in order.

### 4.2. Simulated Data on a 2D Chest-Shaped Tank with a Spatial Prior

**Figure 2:**
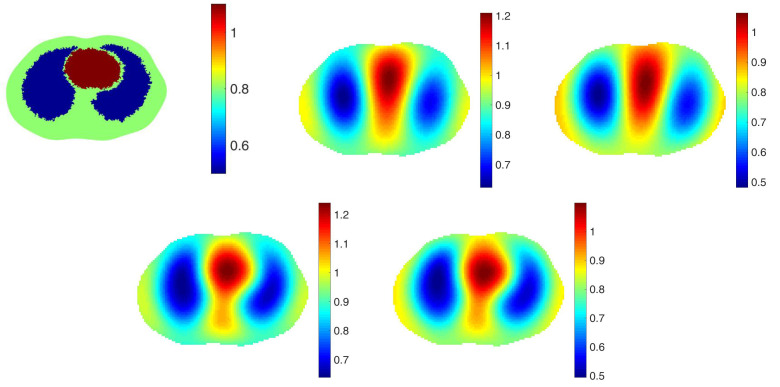
Healthy heart and lungs. From left: Reconstruction of $\gamma^{\mathrm{abs}}$ with no prior, $\gamma^{\mathrm{diff}}$ with no prior, $\gamma_{\mathrm{pr}}^{\mathrm{abs}}$ with the spatial prior, $\gamma^{\mathrm{diff}}$ with the spatial prior. The L2 norms of the difference between those reconstruction and the ground truth, the first figure, are 0.2952, 0.3119, 0.2920 and 0.3085 in order.

**Figure 3:**
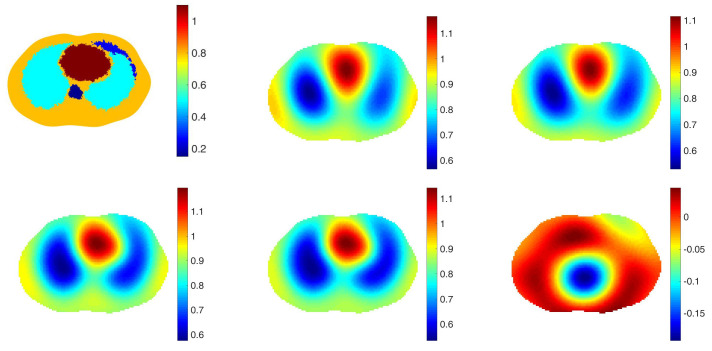
Small pneumothorax. From top left: The true conductivity distribution, reconstruction of $\gamma^{\mathrm{abs}}$ with no prior, $\gamma^{\mathrm{diff}}$ with no prior, $\gamma_{\mathrm{pr}}^{\mathrm{abs}}$ with the spatial prior, $\gamma_{\mathrm{pr}}^{\mathrm{diff}}$ with the spatial prior, a difference image in which the data for the example of healthy heart and lungs in Figure 2 with no pathology is chosen for $\gamma_{\mathrm{ref}}$. The L2 norms of the differences between those reconstructions and the ground truth are 0.3038, 0.3137, 0.3013 and 0.3112 in order.

**Figure 4:**
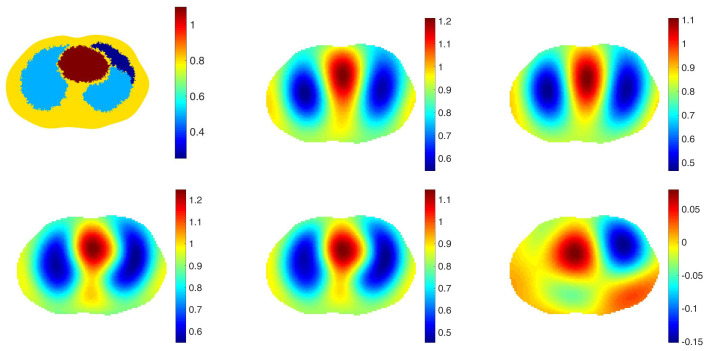
Large pneumothorax. From top left: The true conductivity distribution, reconstruction of $\gamma^{\mathrm{abs}}$ with no prior, $\gamma^{\mathrm{diff}}$ with no prior, $\gamma_{\mathrm{pr}}^{\mathrm{abs}}$ with the spatial prior, $\gamma_{\mathrm{pr}}^{\mathrm{diff}}$ with the spatial prior, a difference image in which the data for the example of healthy heart and lungs in Figure 2 with no pathology is chosen for $\gamma_{\mathrm{ref}}$. The L2 norms of the differences between those reconstructions and the ground truth are 0.3076, 0.3261, 0.3047 and 0.3232 in order.

**Figure 5:**
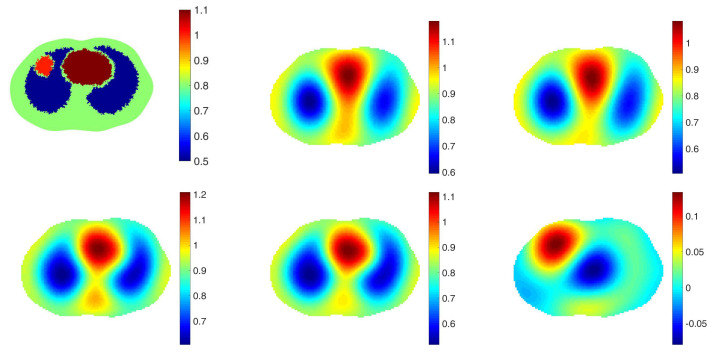
Simulated pulmonary contusion. From left: The true conductivity distribution, reconstruction of $\gamma^{\mathrm{abs}}$ with no prior, $\gamma^{\mathrm{diff}}$ with no prior, $\gamma_{\mathrm{pr}}^{\mathrm{abs}}$ with the spatial prior, $\gamma_{\mathrm{pr}}^{\mathrm{diff}}$ with the spatial prior, a difference image in which the data for the example of healthy heart and lungs in Figure 2 with no pathology is chosen for $\gamma_{\mathrm{ref}}$. The L2 norms of the differences between those reconstructions and the ground truth are 0.2958, 0.3071, 0.2927 and 0.3040 in order.

**Figure 6:**
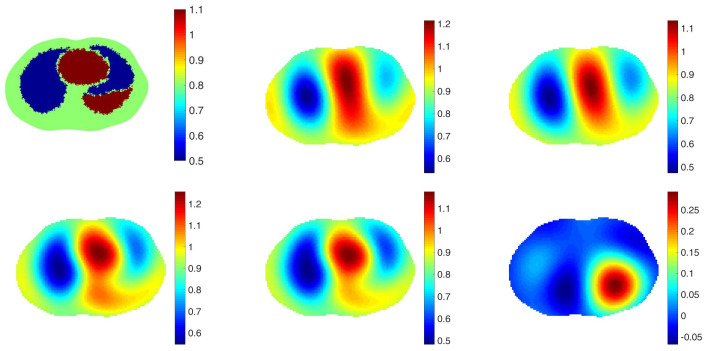
Simulated pleural effusion. From left: The true conductivity distribution, reconstruction of $\gamma^{\mathrm{abs}}$ with no prior, $\gamma^{\mathrm{diff}}$ with no prior, $\gamma_{\mathrm{pr}}^{\mathrm{abs}}$ with the spatial prior, $\gamma_{\mathrm{pr}}^{\mathrm{diff}}$ with the spatial prior, a difference image in which the data for the example of healthy heart and lungs in Figure 2 with no pathology is chosen for $\gamma_{\mathrm{ref}}$. The L2 norms of the differences between those reconstructions and the ground truth are 0.3000, 0.3065, 0.2966 and 0.3029 in order.

The purpose of including a spatial prior in the reconstruction algorithm in the clinical setting is to improve resolution and detection of pulmonary pathology without biasing the reconstruction towards any particular pathology. With this goal, we take the point of view that the spatial prior should represent a normal chest with healthy lungs. To study the effectiveness of this approach, reconstructions from simulated data with 0.1% added noise representing healthy lungs and four different pathologies were computed using a healthy lung prior. In all cases, the correct electrode locations were used in the reconstructions. Four simulated pathologies were studied: a small pneumothorax and a large pneumothorax in the left lung, a contusion in the right lung, and a pleural effusion in the left lung. Reconstructions are found in Figure 2, Figure 3, Figure 4, Figure 5 and Figure 6, respectively. The prior was constructing from the healthy lung case. The conductivity values for each of the organs and pathologies are found in Table 1, and were chosen in agreement with the references [43,44]. In the simulation, data with no inclusions are computed and used for the difference images.

In each example with a pathology, the pathology is clearly visible in the difference image created by subtracting the data from the simulated healthy heart and lung case from the data corresponding to the case with the pathology (the lower right image in each of these figures). This simulates detection of a pathology that occurs while the patient is being monitored, and pre-pathology data is available. The images of $\gamma^{\mathrm{abs}}$ with no prior, $\gamma^{\mathrm{diff}}$ with no prior, $\gamma^{\mathrm{abs}}$ with the spatial prior, $\gamma^{\mathrm{diff}}$ with the spatial prior have more subtle differences from one another. In the case of the small and large pneumothoraces, the left lung is visibly larger, and resistive region near the top of the left lung expands. In the simulation of the pulmonary contusion, the contusion is arguably invisible in the reconstructions, but clearly visible in the difference image in the lower right. In the simulation of the pleural effusion, the effusion is clearly visible in each of the reconstructions, although not well-localized.

The results suggest that the method is best-suited for monitoring for developing pathologies as opposed to detection of a pathology already present with no reference image in which the pathology is absent. We are not assuming that we can collect EIT data on both a healthy and injured lung at the same time. In the case of a contusion, which is caused by blunt-force trauma to the lung, the clinical scenario most relevant here would be monitoring the resolution or healing of the contusion, using the contusion image as reference.

### 4.3. Human Subject Data

We demonstrate the effectiveness of Calderón’s method with accurate shape and electrode modeling and a spatial prior on archival data collected as part of a larger study conducted in accordance with the amended Declaration of Helsinki—Ethical Principles for Medical Research Involving Human Subjects. Data were collected at Children’s Hospital Colorado, Aurora, CO, under the approval of the Colorado Multiple Institutional Review Board (COMIRB) (approval number COMIRB 14-0652). Informed written parental consent and children’s informed assent was obtained from the subject. Data from one 10-year-old male cystic fibrosis patient collected during a routine annual exam is considered here.

**Figure 7:**
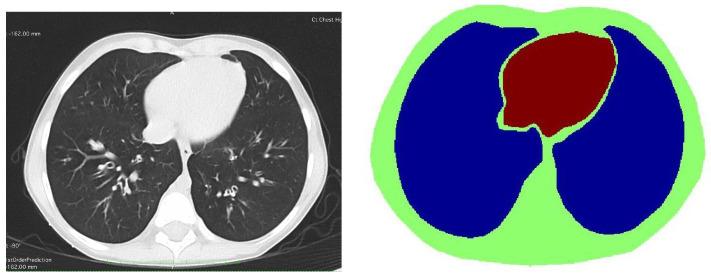
One slice of the CT scan of the subject and the static prior constructed from the CT image.

EIT data were collected during tidal breathing and during breathholding on 22 pediatric Philips EKG electrodes of height 3.33 cm and width 2.22 cm placed around the circumference of the chest. When the electrodes were removed, fiducial markers were placed at the center of each electrode, and a CT scan was performed as part of the subject’s standard care. Bipolar adjacent current patterns were applied with a current amplitude of 2.9 mA on the injecting electrodes. Electrode centers were identified from the inspiratory CT scans with the slices overlaid. The CT scan slide in Figure 7 was used to approximate the boundary shape for the domain and the prior as well as the shape and location of the lungs and heart to create the spatial prior. For the ventilation image sequence, a reference frame was chosen by finding the minimum value of the first component in a principal component analysis (PCA) plot of the data. This frame corresponds to maximum exhalation over the sequence and was the 200th frame. For the breath-holding sequence, the voltage data on each electrode for a fixed current pattern was averaged over all of the frames to create a data set representing a data set averaged over all frames.

**Figure 8:**
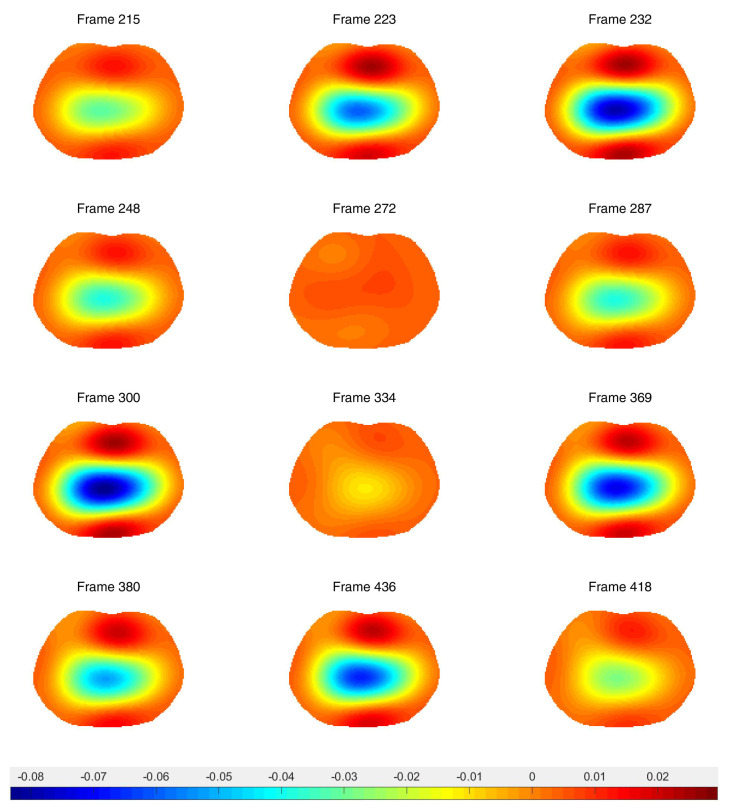
Difference images of ventilation data with no spatial prior included in the algorithm. Frame 200 is used for the reference image. The truncation radius is 1.2.

**Figure 9:**
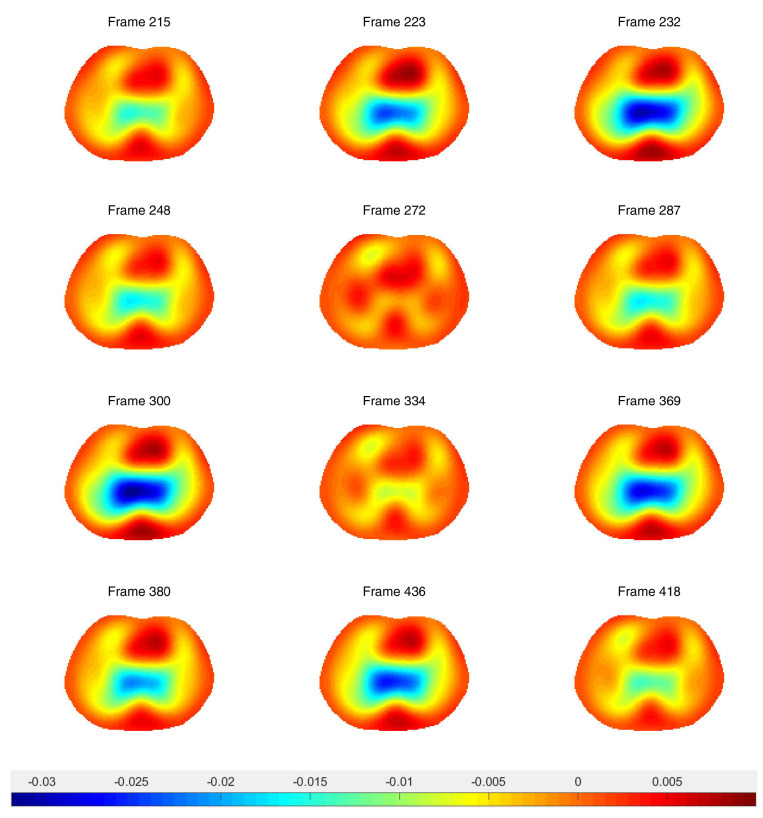
Difference images of ventilation data with the spatial prior included in the algorithm. Frame 200 is used for the reference image. The truncation radius is 1.2 and the outer truncation radius is 3.

**Figure 10:**
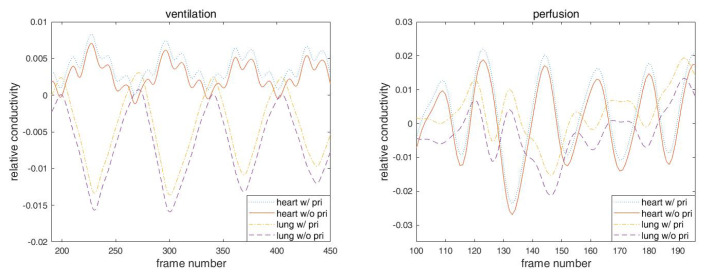
The time trace plots of a pixel in the right lung region and a pixel in the heart region from the reconstructions. Two pixels are marked with squares in the first image of Figure 12. Left is the plot from the ventilation data and the right one is for the breath-holding data. In both plots, from the top to the bottom, each graph represents the time trace of the pixel in the heart with and without a prior, and the time trace of the right lung pixel with and without a prior. The plots of the lung pixels in the perfusion time trace plot are shifted upward by 0.02 in order to better demonstrate the out-of-phase nature.

After running a preliminary difference reconstruction of $\delta^{\mathrm{diff}}$ with no prior, the maximum conductivity value in the region of the heart was assigned to the heart region in the spatial prior, the minimum conductivity value in the region of the lungs was assigned to the lung regions in the spatial prior, and the background conductivity was set to zero, since these are difference images. Difference images of ventilation data with no prior and truncation radius 1.2 are found in Figure 8. While ventilatory changes are clearly visible, the spatial resolution is very poor. Difference images with a spatial prior with an inner truncation radius of 1.2 and outer truncation radius of 3 are found in Figure 9. The spatial resolution has improved when compared to the CT scan and the ventilatory changes are still clearly visible. In Figure 10, time traces of a pixel from the lung region and from the heart region are superimposed both for the no-prior reconstructions and the reconstructions with the static spatial prior.

**Figure 11:**
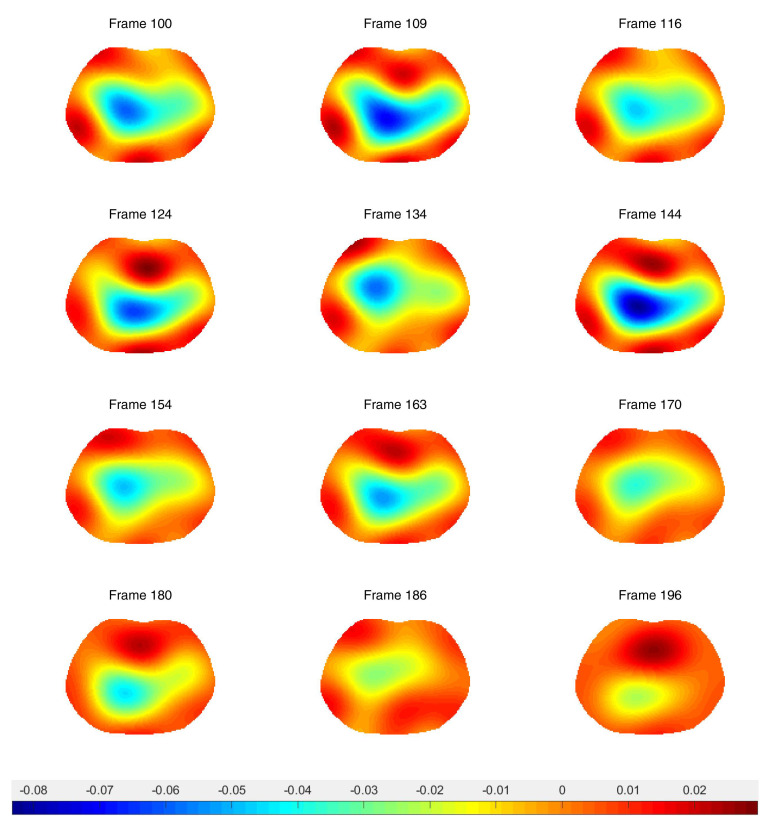
Difference images of breath holding data with no spatial prior included in the algorithm. Averaged voltage data were used for the reference data. The truncation radius is 1.2.

**Figure 12:**
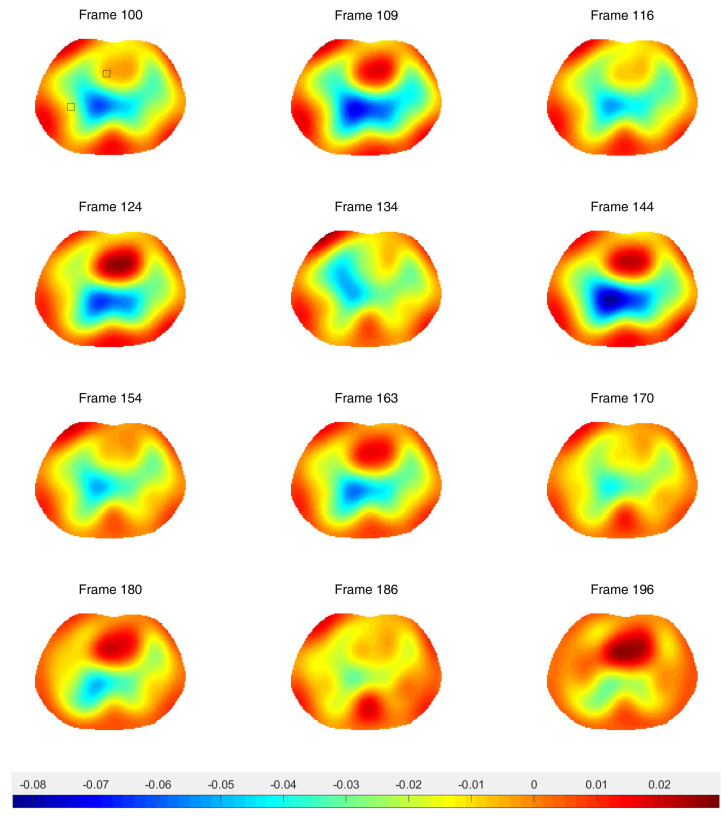
Difference images of breath holding data with the spatial prior included in the algorithm. Averaged voltage data were used for the reference data. The inner truncation radius is 1.2 and the outer truncation radius is 3. The small squares superimposed on the upper left figure indicate the pixels chosen for the time traces in Figure 10.

**Figure 13:**
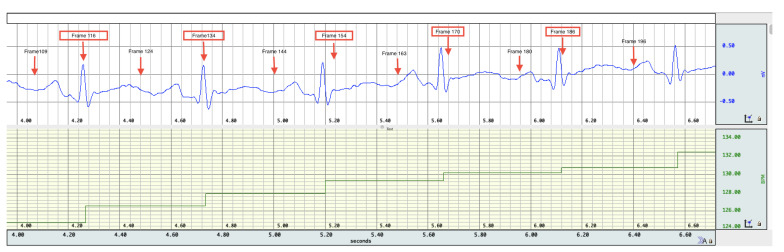
EKG data collected with BIOPAC during breath-holding. Frames from Figure 11 and Figure 12 are annotated on the EKG trace in the upper half of the image. One sees that systole in the EIT images closely coincides with the QRS complex in the EKG data, as would be expected. The average heart rate is found in the lower half of the figure.

Difference images from the breath holding data with no prior and truncation radius 1.2 are found in Figure 11, and reconstructions with the prior are found in Figure 12. In this case, a reference DN map was computed by averaging the voltages over all frames to compute an average reference frame. That is, for each fixed current pattern and each electrode, an average voltage over all frames was assigned to the voltage component in the computed reference data. The plotted images correspond to systole and diastole, as selected manually from the images. A movie of the full reconstruction sequence can be found in the Supplementary Data. The BIOPAC 3-lead EKG data are plotted in Figure 13. Since the ACE 1 system with 22 electrodes collects data at 36.28 frames/s, each frame corresponds to 0.276 s of data. Since start time of the BIOPAC data collection was delayed by 1.06 s, frame 100, the first frame found in Figure 11 and Figure 12, correspond to 3.82 s, and we display the relevant portion of the EKG output. For reference, each small rectangle in the EKG figure represents 0.04 s or 1.45 frames. Frames from Figure 11 and Figure 12 are marked with arrows, and the frames corresponding to systole have red box around them. One sees that systole in the EIT images closely coincides with the QRS complex in the EKG data, as would be expected. The average heart rate can be seen in the lower half of the figure. From the images, one also sees that the heart region is less red when the heart ejects blood during systole, and becomes deeper red during diastole. The lung regions shrink and become less blue during systole as blood enters the lungs and they become more conductive, and they become bluer as the heart fills during diastole, as the conductive blood leaves the lungs and returns to the heart. In Figure 10, we plot the time trace of two pixels which are indicated with squares in the first image of Figure 12. The left plot is the time trace of the ventilation data and the right one is for the breath-holding data. In both plots, each graph represents, from the top to the bottom, the heart region with and without a prior and the lung region with and without a prior. The two plots show the breath and the beating of the heart clearly. The left plot shows frames 190 to 450, demonstrating about 15.5 heart beats and five breath cycles. The right plot shows frames 100 to 196, depicting about 5.5 heart beats and the perfusion in the lung region. In the plot from the breath-holding data, the time trace of the lung pixel and the time trace of the heart pixel are out-of-phase, as is expected.

The full image sequences of reconstructions for Figure 8, Figure 9, Figure 11 and Figure 12 can be found as movies on https://youtu.be/0qxE4Aosmtw, https://youtu.be/oHTjZGnWLKQ, https://youtu.be/1PP7c9E17sM, and https://youtu.be/q_yugxxhdHM all accessed on 13 August 2021, in order.

**Figure 14:**
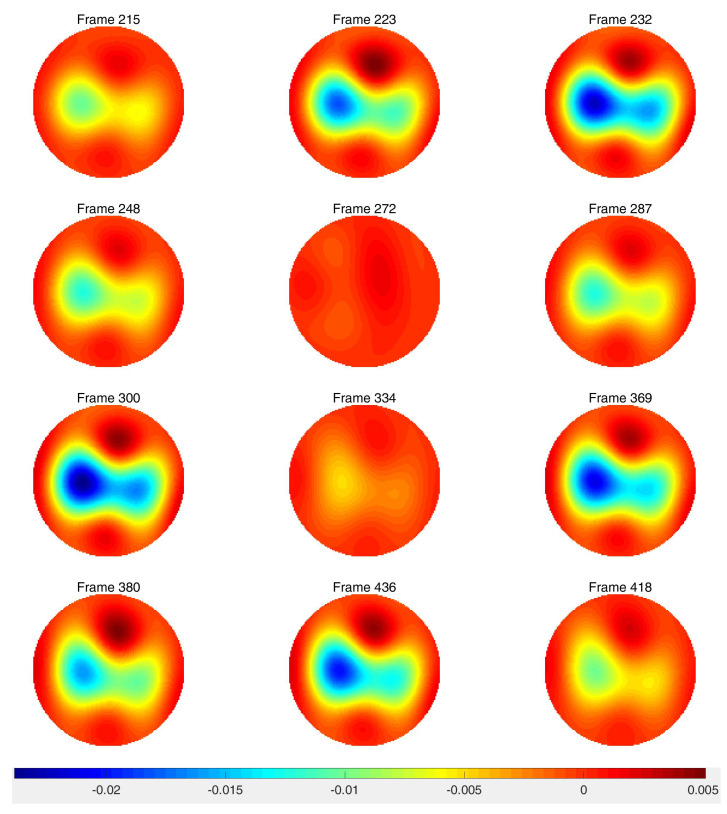
Difference images of ventilation data with modeling the domain to be a circle. No prior is added. Frame 200 is used for the reference image. The truncation radius is 1.2.

**Figure 15:**
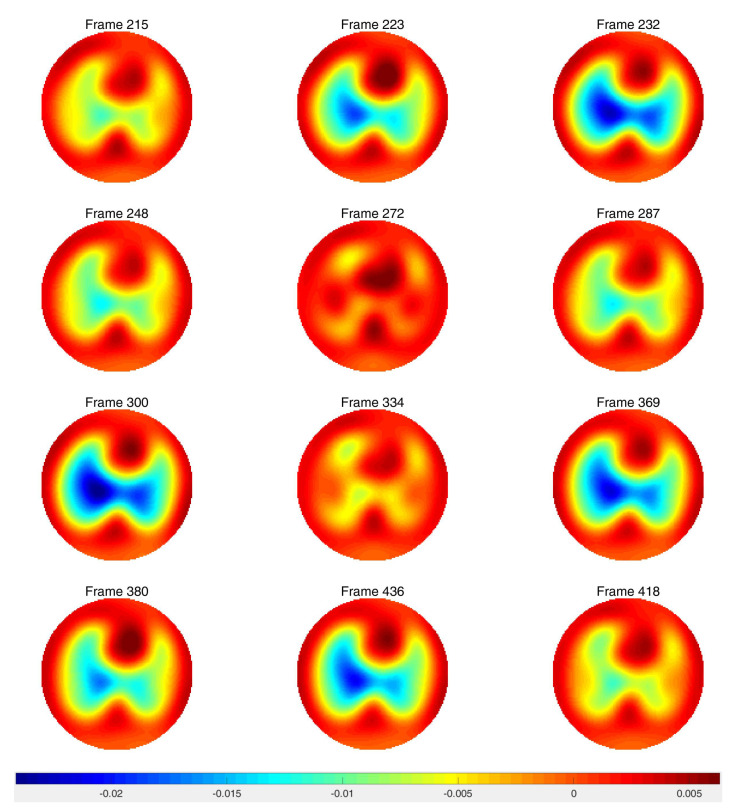
Difference images of ventilation data with modeling the domain to be a circle. A spatial prior is added. Frame 200 is used for the reference image. The truncation radius is 1.2.

Reconstructions of ventilation computed on a circular domain of the same perimeter as the true domain are included in Figure 14 and Figure 15. These images show separation between the lungs, as may be familiar to the reader for EIT reconstructions, but it is an artifact of the circular domain, which has the effect of pulling the targets toward the boundary, as has been previously reported in Figure 14 and Figure 15 for Calderon’s method. Due to the very close proximity of the lungs to each other near the heart (see the CT scan in Figure 7), the lungs should not show a large separation. In fact, the reconstructions from the prior show an indentation near the bottom (at the location of the spine), which is to be expected in a difference image, and this is further evidence of the improved resolution with the prior.

## 5. Conclusions

In this work, Calderón’s method in two dimensions was implemented with correct electrode positions for experimental and simulated data and shown to yield improved difference and absolute images compared to when the electrodes are assumed to be equally spaced. In addition, a spatial prior was included in the reconstruction algorithm and demonstrated to improve spatial resolution and accuracy of conductivity values. Examples from human data demonstrate the method’s ability to image conductivity changes due to ventilation and pulsatile pulmonary perfusion.

## Figures and Tables

**Table 1:** Conductivity values for the simulated data by organ.

Organ/Region	Conductivity (S/m)	True $\delta^{\mathrm{abs}}$ Value
Background	0.8	0
Heart	1.1	0.3
Lung	0.5	−0.3
Spine	0.15	−0.65
Pneumothorax	0.25	−0.55
Contusion	0.6	−0.2
Effusion	1.0	0.2

## Supplementary Materials

The original files for Figure 8, Figure 9, Figure 10 and Figure 11 are available online at https://www.mdpi.com/article/10.3390/s21165635/s1.
